# Neuro-imaging in intracerebral hemorrhage: updates and knowledge gaps

**DOI:** 10.3389/fnins.2024.1408288

**Published:** 2024-05-09

**Authors:** Mary Penckofer, Khuram S. Kazmi, Jesse Thon, Daniel A. Tonetti, Casey Ries, Swarna Rajagopalan

**Affiliations:** ^1^Cooper Medical School of Rowan University, Camden, NJ, United States; ^2^Department of Neuroradiology, Cooper University Health Care, Camden, NJ, United States; ^3^Department of Neurology, Cooper University Health Care, Camden, NJ, United States; ^4^Department of Neurosurgery, Cooper University Health Care, Camden, NJ, United States; ^5^Department of Radiology, Cooper University Health Care, Camden, NJ, United States

**Keywords:** intracerebral hemorrhage, magnetic resonance imaging, neuroimaging, computed tomography, hematoma

## Abstract

Intracerebral hemorrhage (ICH) is characterized by hematoma development within the brain’s parenchyma, contributing significantly to the burden of stroke. While non-contrast head computed tomography (CT) remains the gold standard for initial diagnosis, this review underscores the pivotal role of magnetic resonance imaging (MRI) in ICH management. Beyond diagnosis, MRI offers invaluable insights into ICH etiology, prognosis, and treatment. Utilizing echo-planar gradient-echo or susceptibility-weighted sequences, MRI demonstrates exceptional sensitivity and specificity in identifying ICH, aiding in differentiation of primary and secondary causes. Moreover, MRI facilitates assessment of hemorrhage age, recognition of secondary lesions, and evaluation of perihematomal edema progression, thus guiding tailored therapeutic strategies. This comprehensive review discusses the multifaceted utility of MRI in ICH management, highlighting its indispensable role in enhancing diagnostic accuracy as well as aiding in prognostication. As MRI continues to evolve as a cornerstone of ICH assessment, future research should explore its nuanced applications in personalized care paradigms.

## 1 Introduction

Intracerebral hemorrhage (ICH) is characterized by hematoma development within the brain’s parenchyma. In the United States alone, it contributes to roughly 10–20% of the 795,000 annual stroke cases (Rajashekar and Liang, 2023; Unnithan et al., 2023). ICH can be broadly classified into primary and secondary forms, with primary ICH accounting for about 80% of cases, while secondary ICH makes up the remaining 20% (Macellari et al., 2014; Jain et al., 2021). Primary ICH stems from underlying small vessel diseases such as hypertension and cerebral amyloid angiopathy (CAA), whereas secondary ICH can result from various factors such as hemorrhagic conversion of acute ischemic stroke (AIS), vascular malformations, or other structural anomalies (Macellari et al., 2014; Raposo et al., 2023). This review delineates the role of magnetic resonance imaging (MRI) in ICH, including its use for diagnosis, treatment, and prognostication.

## 2 Need for imaging in ICH and urgency

ICH is associated with high rates of severe disability and mortality, including an approximately 40% mortality rate within 1 month and a combined severe disability and mortality rate of up to 75% within 1 year (van Asch et al., 2010; Jain et al., 2021). Given these data on the severity of the natural history of ICH, emergent evaluation is critical. Early identification enables initiation of ultra-early treatment to prevent hematoma expansion and mitigate neuroinflammation. The potential of reduced morbidity and mortality associated with ICH through timely recognition and intervention has led to an increasing emphasis on its prompt diagnosis (Li et al., 2024).

Initial imaging for ICH is predicated on non-contrast head computed tomography (NCCT), commonly performed in the emergency department. CT or MR angiography may be used to identify risk of hematoma expansion and vascular causes, while MRI is also effective in identifying other structural causes of ICH (Hemphill 3rd et al., 2015). Once an ICH is visualized using neuroimaging, early interventions via management of airway, hemostasis, seizures, hypertension, intracranial hypertension, hyperglycemia, fever, and surgical intervention are critical as they can contribute to decreased rates of morbidity and mortality (Morotti and Goldstein, 2016). Rapid imaging is crucial for swift diagnosis. While both CT and MRI are highly sensitive in diagnosing ICH, a NCCT is typically the first-line diagnostic tool due to its ability to differentiate between ischemic strokes and ICH, wider availability, shorter scanning time and patient factors such as clinical instability, presence of pacemaker or claustrophobia (Macellari et al., 2014; Morotti and Goldstein, 2016; Fandler-Höfler et al., 2023).

## 3 Role of MRI in ICH diagnosis

MRI demonstrates sensitivity, specificity, and overall accuracy, approaching nearly 100%, for diagnosing ICH in hyperacute and acute settings (Fiebach et al., 2004; Romanova et al., 2014). MRI, specifically echo-planar gradient-echo (GRE) or susceptibility-weighted imaging (SWI) sequences, are particularly useful in the hyperacute (less than 24 h of symptom onset) setting of ICH because as time progresses, the hemorrhage can obscure the underlying brain parenchyma, and this occurs to a greater degree after 24 h. MRI also has higher diagnostic accuracy for chronic ICH when compared to CT (Linfante et al., 1999; Kidwell et al., 2004; Macellari et al., 2014). However, despite this, MRI should not replace CT as the primary imaging method due to its greater expediency, which is crucial for promptly detecting ICH.

**Figure 1:**
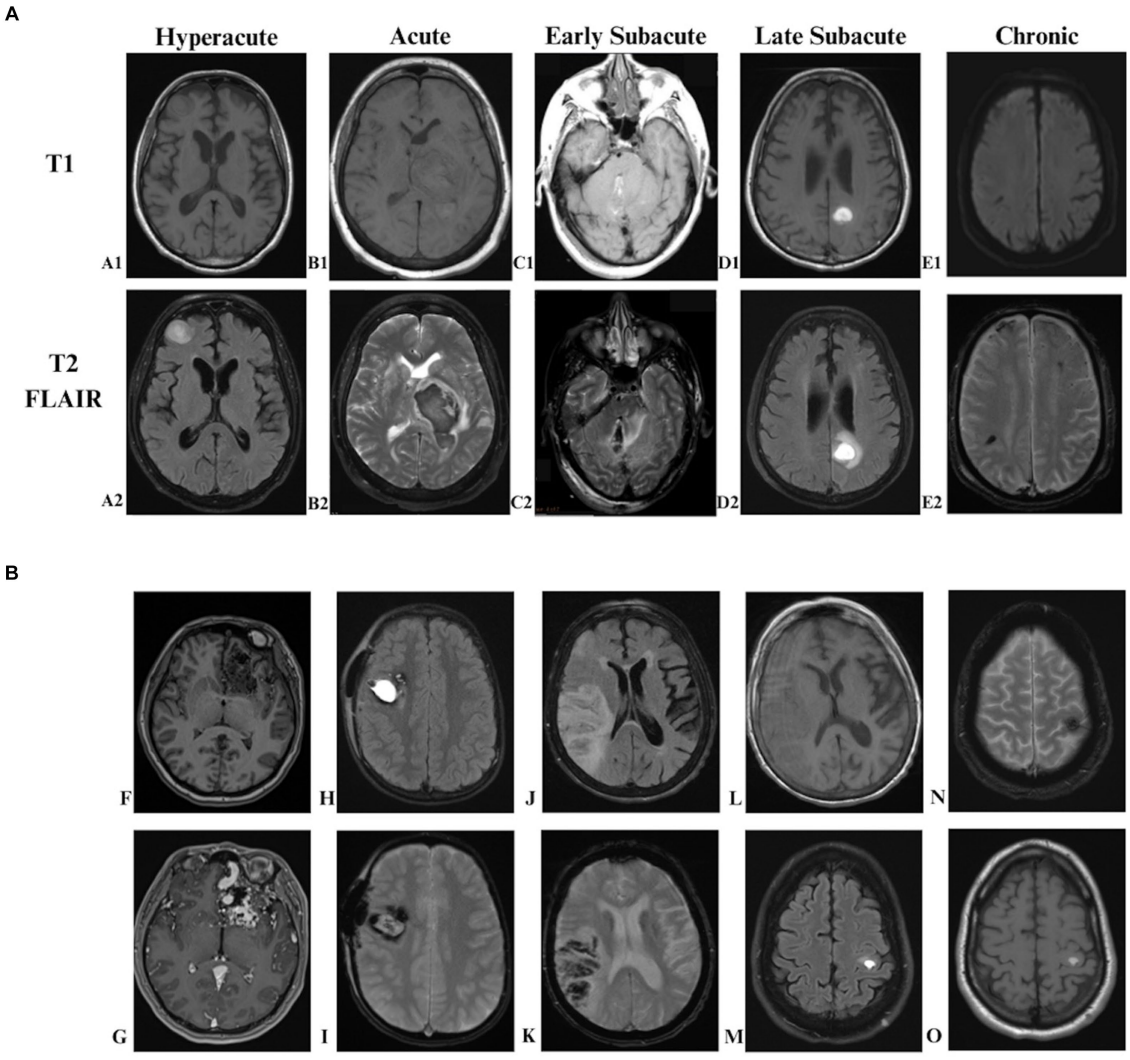
ICH stages of blood over time on MRI and common Etiologies of ICH on MRI. **(A)** Stages of blood in ICH: **(A1)** ICH hyperacute blood on T1, **(A2)** ICH hyperacute blood on T2 FLAIR, **(B1)** ICH acute blood on T1, **(B2)** ICH acute blood on T2 FLAIR, **(C1)** ICH early subacute blood on T1, **(C2)** ICH early subacute blood on T2 FLAIR, **(D1)** ICH late subacute blood on T1, **(D2)** ICH late subacute blood on T2, **(E1)** ICH chronic blood on T1. **(E2)** ICH chronic blood on GRE. **(B)** Common ICH etiologies on MRI: **(F)** AVM on T1 Pre-Contrast, **(G)** AVM on T1 Post-Contrast, **(H)** Cavernous hemangioma on T2 FLAIR, **(I)** Cavernous hemangioma on GRE, **(J)** Hemorrhagic conversion of ischemic stroke on T2 FLAIR, **(K)** Hemorrhagic conversion of ischemic stroke on GRE, **(L)** Hemorrhagic conversion of ischemic stroke on T1, **(M)** Hemorrhagic metastasis on T2 FLAIR, **(N)** Hemorrhagic metastasis on GRE, **(O)** Hemorrhagic metastasis on T1. ICH = Intracerebral hemorrhage, FLAIR = fluid-attenuated inversion recovery, GRE = gradient-echo, AVM = arteriovenous malformation.

MRI is a useful tool in determining the acuity of ICH, especially in instances where the ICH is composed of hemorrhagic components of different ages, or when the patient has had multiple ICHs. The findings on MRI are dependent on the age of the hemorrhage. As a hematoma ages, hemoglobin undergoes various transformations, transitioning through oxyhemoglobin, deoxyhemoglobin, methemoglobin, and ultimately leading to the breakdown of red blood cells (RBCs) into ferritin and hemosiderin (Bradley, 1993). As the hemoglobin breaks down from oxyhemoglobin to the rest of the RBC products, it transitions from diamagnetic material, having no unpaired electrons, to paramagnetic material, having unpaired electrons. The intensity seen on MRI depends on whether unpaired electrons are present, how many unpaired electrons there are, and the location of the RBC products. The phases of ICH (see Table 1) can be classified as hyperacute (< 24 h of the hemorrhage, intracellular oxyhemoglobin, Figure 1A, Images A1,A2), acute (1–3 days, intracellular deoxyhemoglobin, Figure 1A, Images B1,B2), early subacute (3–7 days, intracellular methemoglobin, Figure 1A, Images C1,C2), late subacute (7–28 days, extracellular methemoglobin, Figure 1A, Images D1,D2), or chronic (>1-month, extracellular ferritin and hemosiderin, Figure 1A, Images E1,E2) (Bradley, 1993; Weerink et al., 2023).

**Table 1:** Stages of hemorrhage on MRI.

	T1 Sequence MR	T2 Sequence MR	Gradient-echo sequence MR
Hyperacute (<24 h)	Hypointense/isointense	Isointense/hyperintense center with peripheral hypointensity and hyperintense rim of vasogenic edema	Marked hypointensity
Acute (1–3 days)	Isointense/slightly hypointense	Hypointense with hyperintense rim	Marked hypointensity
Early subacute (3–7 days)	Hyperintense	Hypointense	Hypointense
Late subacute (7–28 days)	Hyperintense	Hyperintense	Hypointense
Chronic (>1 month)	Hypointense	Hypointense	Hyperintense/Isointense core with hypointense rim

## 4 Role of MRI in identifying an ICH etiology

MRI is useful in identifying primary causes of ICH, such as small-vessel disease (SVD) or cerebral amyloid angiopathy (CAA). In particular, findings of lobar macrohemorrhage, exclusively cortical microbleeds (CMBs), cortical superficial siderosis (cSS), and a multispot pattern of white matter hyperintensities have been associated with CAA, whereas CMBs in subcortical locations and basal ganglia white matter hyperintensities may indicate SVD as the ICH etiology (Charidimou et al., 2016, 2022). Making the distinction between CAA and SVD is essential, as it has implications on risk of ICH recurrence, progression, and decision-making regarding the safety of antithrombotic treatments (Charidimou et al., 2019).

MRI plays a critical role in identifying secondary causes of ICH as well. These include structural vascular lesions such as arteriovenous malformations (AVMs), cavernomas, or dural arteriovenous fistulae. Other readily apparent secondary causes of ICH diagnosed predominantly via MRI include hemorrhagic conversion of AIS, cerebral neoplasms, along with cerebral venous thrombosis (CVT), arterial dissection and non-atheromatous vasculopathies such as moyamoya disease, vasculitis, reversible cerebral vasoconstriction syndrome (RCVS) and infective endocarditis (IE) (Wijman et al., 2010; Macellari et al., 2014; Morotti and Goldstein, 2016; Fandler-Höfler et al., 2023; Raposo et al., 2023). In addition, MRI can even be used to aid diagnosing IE in individuals with silent emboli, without neurologic symptoms (Habib et al., 2015). Given its utility in identifying an etiology for ICH, MRI is recommended in all patients without a clear macrovascular cause of ICH identified on a CTA (Greenberg et al., 2022).

MRI is the most sensitive and specific method for identifying cerebral cavernous malformations, which often exhibit a distinct “popcorn” appearance on T2-weighted imaging, with central hyperintensity indicating recent bleeding and a surrounding hypointense halo indicative of hemosiderin from prior bleeding events (Figure 1B, Images H,I). In cases of CVT, contrast-enhanced MR venography provides detailed visualization of thrombosed segments within the venous sinus, showing strong correlation with conventional digital subtraction angiography (DSA) findings and distinguishing anatomical variations, such as hypoplastic sinuses, from CVT. CVT, which is defined as a thrombus in a venous sinus, superficial intracranial vein, or deep intracranial vein (Oliveira et al., 2022) can also be visualized on MRI. This is due to the fact that patent dural sinuses typically appear as a flow void – a signal loss that occurs within moving fluids. Meanwhile CVTs can be recognized best on T2 or fluid-attenuated inversion recovery (FLAIR) sequences as an absence of a flow void (Chiewvit et al., 2011; Oliveira et al., 2022). MRI is also useful in identifying AVMs, where clusters of hypointense vascular channels and enlarged draining veins (pre-contrast, Figure 1B, Image F) enhance following contrast administration (post-contrast, Figure 1B, Image G).

Diffusion-weighted imaging (DWI) sequences of MRI play a crucial role in the evaluation of ICH, as they can help to differentiate between AIS with hemorrhagic conversion and primary ICH. Central hyperintensity on DWI can be seen in both AIS as well as hyperacute hematomas, and these etiologies can appear very similar on T2 weighted imaging. However, using FLAIR sequences can allow for the visualization of the peripheral hypointense rim that is present in hyperacute hematoma and not in AIS (Kang et al., 2001). AIS with hemorrhagic conversion may also have a mixed appearance on DWI sequence, and have microbleeds on GRE or SWI, as opposed to a more homogenous appearance of ICHs (Bradley, 1993).

## 5 Role of MRI in ICH treatment

ICH results in blood–brain barrier (BBB) disruption and parenchymal cellular swelling, contributing to perihematomal edema (PHE). This can cause brain tissue compression, intracranial hypertension, herniation and death. The fluid-attenuated inversion recovery (FLAIR) sequence of the MRI can be used to quantify the volume of PHE, which appears hyperintense (Urday et al., 2015; Ironside et al., 2019). CT can be used for this purpose, however both manual and automated quantification are limited by interference of leukoariosis and cerebrospinal fluid, which are of similar Hounsfield unit densities (Urday et al., 2015). While PHE evolution may correlate with functional outcomes, minimally invasive surgery (MIS) for hematoma evacuation has been shown to be associated with decreased clot burden, PHE, and more recently improved functional outcomes (Mould et al., 2019; Hieber et al., 2023; Kellner and Mocco, 2023; Pradilla et al., 2024). The degree of clot evacuation by MIS has correlated with a decrease in pericavity edema (PCE) and PCE remained static following MIS ICH evacuation in one study (Horowitz et al., 2022).

Patients with ICH may also be at risk of AIS and other cardiovascular events. When antithrombotic or anticoagulant medications are considered for secondary stroke prevention, risk of hemorrhage needs to be carefully weighed against the risk of ischemic and vaso-occlusive diseases (Greenberg et al., 2022). MRI can identify ICH causes that have a heightened risk of ICH recurrence such as CAA, or IE hence can provide useful information when making these treatment decisions. In patients with IE, MRI can also aid in lesion characterization such as identification of abscesses, mycotic aneurysms, lobar hematomas and territorial strokes prior to surgical evaluation for valve surgery (Chakraborty et al., 2019).

If the initial MRI does not reveal an underlying etiology of the ICH, a follow-up MRI could be useful to alter future management. Limited literature suggests that the yield of repeat MRI to detect a secondary lesion varies between 0 to 16% of patients with spontaneous ICH, with secondary lesions typically found to be tumors or cavernomas (Mouchtouris et al., 2021; Wilson et al., 2023). Studies evaluating outpatient follow-up MRI in this setting have included studies performed any time from 1 day to 2 years post-ICH, and the optimal timing of this repeat imaging remains unclear (particularly given the complex evolution of blood products discussed above) (Mouchtouris et al., 2021; Wilson et al., 2023). Future research is required to determine optimal timing for follow-up MRI scans, as well as to identify the specific patient cohorts who would benefit most from such assessments. This may involve considering factors such as the location of ICH and other pertinent imaging characteristics. Such efforts may enhance diagnostic accuracy while minimizing unnecessary testing and associated costs.

## 6 Role of MRI in ICH risk stratification and prognostication

In the acute phase, an MRI can provide more detailed information on the severity of ICH-related injury such as hematoma volume, the degree of surrounding edema and midline shift, assisting in neuroprognostication. Involvement of critical parts of the ascending reticular activating system, caudate nucleus, thalami or diffuse damage impairing network connectivity identified by MRI, can aid in prediction of recovery from disorders of consciousness caused by ICH (Rohaut et al., 2019). Task-based and resting-state functional MRI can reveal cognitive motor dissociation in patients that appear to be unresponsive on bedside examination and predict re-emergence of consciousness (Edlow et al., 2021).

MRI can also aid in individual ICH risk stratification. In patients with ICH, features suggestive of CAA on MRI are associated with the highest ICH recurrence (Greenberg et al., 2022). MRI findings suggestive of cerebral small vessel disease (SVD) and CMBs strongly correlate with poorer functional and cognitive outcomes, an increased risk of ICH recurrence and increased long-term mortality (Greenberg et al., 2022). Notably, in patients with lobar ICH, the presence of cSS, a marker of hemorrhagic risk in CAA, has been associated with higher odds of hematoma expansion and an independent biomarker of poor prognosis (Boulouis et al., 2016; Sporns et al., 2021). The presence of cSS is also associated with a higher recurrence risk in patients with lobar ICH, worse cognitive trajectories, and a higher incidence of post-ICH dementia. In patients with AIS, CMBs detected on GRE or SWI are associated with higher rates of hemorrhagic transformation (Dar et al., 2018). In addition to identification of macrovascular causes of ICH, MRI can identify small intraventricular hemorrhages that may not be detected on CT, allowing a superior estimation of ICH recurrence (Romanova et al., 2014).

PHE, which represents the inflammatory and cytotoxic responses of the tissue surrounding the ICH and can be a quantifiable marker of secondary brain injury (SBI). Variations in pathophysiological mechanisms could affect temporal patterns of PHE formation. While it has been suggested that peak PHE volume typically occurs between 2 to 3 weeks after ICH, observations suggest that PHE can continue to progress for up to 21 days following the onset of ICH (Urday et al., 2015; Ironside et al., 2019; Chen et al., 2021). PHE volume on admission in small ICHs, and PHE increase at 72 h, have been correlated with worse functional outcomes in small studies, with variable methods of PHE quantification (Sansing et al., 2011; Appelboom et al., 2013; Urday et al., 2015).

DWI sequences can also detect small remote DWI hyperintensities in patients with ICH (Xu et al., 2017). Studies have shown a correlation between aggressive blood pressure reduction, systolic blood pressure variability, and the occurrence of DWI MRI lesions, suggesting that acute disruptions in blood pressure autoregulation may contribute to their development (Kidwell et al., 2017). These lesions have been associated with conditions such as SVD and CAA, and their pathogenesis may involve additional factors related to microangiopathy and characteristics specific to ICH (Wu et al., 2015; Murthy et al., 2020; Xu et al., 2022) Despite being often subclinical and unidentifiable on CT, the presence of punctate ischemic DWI lesions increases the risk of subsequent ischemic stroke by 2.5 times, and may be associated with worse long-term outcomes (Murthy et al., 2021). Identifying these DWI lesions through MRI could provide valuable insight for stratifying patients based on potential outcomes, and could even guide acute blood pressure targets, highlighting the utility of MRI in assessing patient prognosis.

Finally, previous literature has utilized MRI in cases of ICH and IVH to stratify patients with the van Swieten scale (vSS) to grade severity of leukoaraiosis via the Fazekas Score (FS) with severe leukoaraiosis defined as FS > 3 or deep FS 2 to 3. Patients with this definition of severe leukoaraiosis were found to have persistently poor outcomes 1 year after their hemorrhagic event. This exemplifies another utilization of MRI for the long term prognostication of ICH patients (Shah et al., 2022).

## 7 Conclusion

MRI is a pivotal tool in the evaluation of ICH. It has a very high sensitivity, specificity, and accuracy in diagnosing ICH, particularly in the hyperacute stage. Beyond its hyperacute utility, MRI can also be utilized to determine ICH age and differentiation of underlying causes, thereby influencing disease-specific treatment strategies, and offering prognostic value. As MRI continues to serve as a cornerstone of ICH assessment and becomes standard of care globally, future studies are needed to assess its use in individualized clinical scenarios, such as the diagnostic value of performing serial studies and the utility of MRI in guiding specific medical and procedural interventions.

## Author contributions

MP: Conceptualization, Data curation, Methodology, Writing – original draft, Writing – review & editing. KK: Writing – original draft, Writing – review & editing. JT: Supervision, Visualization, Writing – review & editing. DT: Investigation, Supervision, Writing – review & editing. CR: Conceptualization, Data curation, Writing – review & editing. SR: Conceptualization, Supervision, Writing – original draft, Writing – review & editing.
